# Protective effects of antidepressants against ulcerogenic agents: insights from nonclinical studies

**DOI:** 10.1007/s10787-026-02240-3

**Published:** 2026-04-22

**Authors:** Caroline Stringari, Heloisa Stringari, Thiago Farias de Queiroz e Silva, Caio Henrique Willrich, Luisa Mota da Silva

**Affiliations:** 1 Pharmaceutical Sciences Graduate Program, University of Itajai Valley, Itajai, SC Brazil; 2 Medicine course, Health school, University of Itajai Valley, Itajai, SC Brazil; 3 Department of Pharmacology, Federal University of Santa Catarina, Florianópolis, SC Brazil

**Keywords:** Serotonin, Gastric ulcers, Fluoxetine, Imipramine, Acute ulcers model

## Abstract

**Introduction:**

There is a marked overlap among the neuronal pathogenetic pathways involved in ulcer genesis and depression, and antidepressants can also exert a protective effect against gastric ulcers.

**Objective:**

To summarize and critically discuss non-clinical evidence about the gastroprotective potential of antidepressants.

**Methodology:**

Articles related to the survey were searched on PubMed, ScienceDirect, MedLine, and Lilacs, identifying 163 publications from 1974 to 2024, after reviewing 27 articles were selected.

**Results:**

All experimental research on the antiulcer potential of antidepressants has used acute gastric ulcer models, mainly ethanol- and indomethacin- induced ulcers. Tricyclic antidepressants, such as amitriptyline, are the most researched and have shown significant gastroprotective effects, reducing ulcer size and severity, and inhibiting NOX2 and NOX4 (enzymes from the NADPH oxidase family) expression in gastric mucosa. Selective serotonin reuptake inhibitors also exhibit gastroprotective effects. Fluoxetine decreased ulcer size in a stress and indomethacin model while increasing antioxidant production and decreasing inflammation in indomethacin-induced ulcers, but one study found that fluoxetine had a greater gastroprotective effect in male rodents than in females, highlighting the need for further research on sex differences. Mirtazapine and duloxetine have also shown gastroprotective benefits. Mirtazapine reduced ulcer size in an indomethacin model. In a stress and indomethacin model, duloxetine reduced ulcer size while also decreasing NOX1 and NOX4 expression. The mechanisms underlying the gastroprotective effects of antidepressants involve peripheral reduction of mucosal oxidative stress and central modulation of vagal stimulus and an increase in noradrenergic and serotonergic neurotransmission.

**Conclusions:**

Overall, the trials examined show that antidepressants are effective in preventing ulcers and may be useful in protecting against gastric ulcer development, especially in people with depression.

**Supplementary Information:**

The online version contains supplementary material available at 10.1007/s10787-026-02240-3.

## Introduction

Peptic ulcers are necrotizing lesions on the gastrointestinal tract (GI), particularly the stomach and proximal duodenum (Graham 2014). Although the occurrence of lesions is associated with an imbalance between aggressive and protective agents of the gastric mucosa, exogenous factors such as Helicobacter pylori infection and prolonged use of non-steroidal anti-inflammatory drugs (NSAIDs) are among the major risk factors, exposing the deleterious action of gastric acid and pepsin on the gastric mucosa (Lanas and Chan 2017; Wu et al. 2021). Gastric ulcers impair the quality of life of their patients, causing epigastric discomfort, a burning feeling, lack of appetite, vomiting, and indigestion (Ramakrishnan, 2007).

Given the role of gastric acid and pepsin secretion in the pathophysiology of gastric ulcers, the current treatment is based on gastric acid-suppressing medications, including histamine receptor antagonists (H2-RAS) (ranitidine, cimetidine, and famotidine), inhibitors of proton pump inhibitors (PPIs) drugs (omeprazole, pantoprazole, and lansoprazole), and, more recently, potassium-competitive acid blockers (P-CABs) (vanoprazan) (Yuan, 2006; Zhou, 2012; Murakami, 2016). However, although playing an important role in treatment, the poor quality of healing of gastric lesions caused by PPIs use promotes the recurrence of gastric ulcers (Kamada, 2020), necessitating the development of novel therapeutic techniques.

Before the clinical use of PPIs, Berardi and Caplan (1983) reviewed tricyclic structures for treating peptic ulcer disease, highlighting clinical studies that showed tricyclic antidepressants (TCAs) such as trimipramine maleate and doxepin hydrochloride to be effective in the treatment of peptic ulcer disease. TCAs are regaining status as agents for the treatment of peptic ulcer disease, according to Ries et al. (1984), who reviewed clinical studies that showed that TCAs reduce gastric acid secretion (most likely due to their well-known anticholinergic properties), while in vitro studies showed potent H1- and H2-receptor blocking activities for these agents. Furthermore, the authors proposed that, in addition to their effects on acid output, TCAs’ analgesic and antidepressant properties may be beneficial in certain ulcer patients. Following that, various non-clinical investigations were conducted to investigate TCAs’ anti-ulcer potential and mechanism of action, which were reviewed and discussed here.

Besides TCAs, other classes of antidepressants have been shown to exert protective effects against ulcerogenic agents, including non-steroidal anti-inflammatory drugs (NSAIDs) and stress-induced gastric damage, and were included in this review. However, it is important to note that epidemiologic studies show that selective serotonin reuptake inhibitors (SSRIs) are associated with roughly doubled odds of upper gastrointestinal (GI) bleeding, mainly with the concurrent use of NSAIDs, anticoagulants, and antiplatelet agents (Andrade et al., 2010).

The mechanisms underlying the gastroprotective effects of antidepressants are complex and multifaceted, involving modulation of gastric acid secretion, antioxidant activity, and central nervous system influences and in the last five decades several non-clinical studies have shown that antidepressants reduce the severity of gastric lesions, promoting gastroprotection with this effect related to a decrease in the volume and acidity of gastric secretion, and conferring beneficial effects on gastric mucosal protection together with central nervous system (CNS) effects.

In addition, the intricate interplay between the gut and the brain, often referred to as the gut-brain axis, has garnered significant attention in recent years, revealing the complex bidirectional communication network that influences gastrointestinal function and overall health. In this context, the modulation of the gut-brain axis by antidepressants antidepressants, which primarily target the CSN, may contribute to their ability to mitigate gastric damage.

In this context, the purpose of this review is to systematically evaluate and summarize the existing non-clinical evidence on the gastroprotective effects of antidepressants, providing a comprehensive overview of their potential mechanisms of action and highlighting the novel insights they may bring to the prevention and treatment of peptic ulcers. By synthesizing the current knowledge on this topic, this review aims to identify areas for future research and potential therapeutic applications, ultimately contributing to the development of new strategies for managing peptic ulcer disease and improving patient outcomes.

## Methodology

This review aimed to map the existing literature on the gastroprotective effects of antidepressants in nonclinical models of gastric ulceration by employing approaches for identifying, selecting, and critically reviewing scientific research. In this way, the study followed the methodology steps based on Whittemore and Knaf’s well-founded proposals: identification of the problem; literature research; data extraction from studies; critical evaluation of studies; interpretation of results; and evidence synthesis (Whittemore, 2005).

The review question was, “What is the experimental evidence for the gastroprotective effect of antidepressants?”

**Fig. 1:**
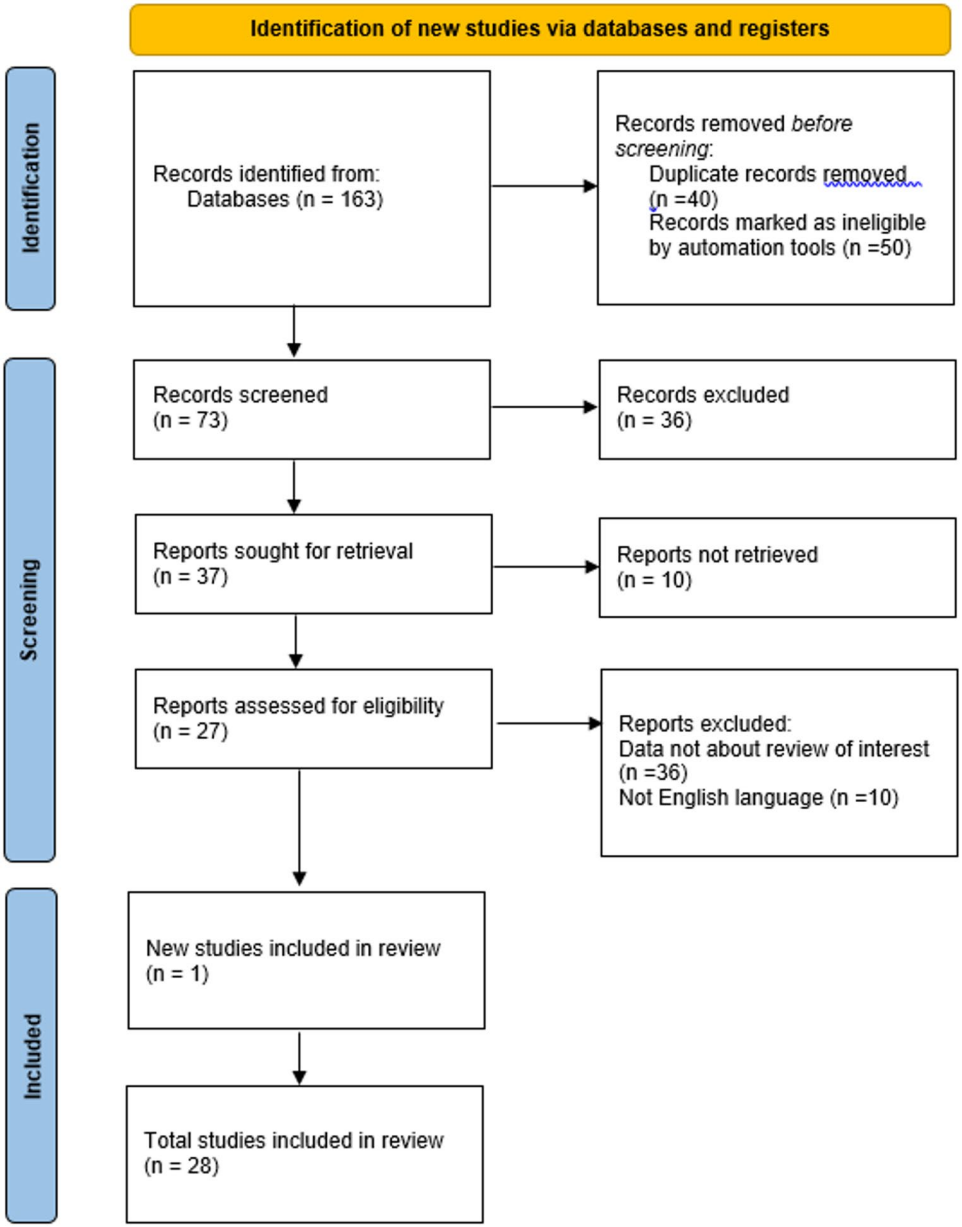
The PRISMA Flow Diagram depicts the article selection process, which includes identification, screening, eligibility, and inclusion stages. Initially, 163 items of interest were discovered, but 40 were deleted for being duplicates, 50 for being published more than 50 years ago (marked as ineligible by an automation tool), 36 for not being of interest for review, and 10 for not being in English, remaining 27 articles for review. After 1 new study was included in the review, resulting in 28 articles were reviewed

The literature search was conducted using the following databases: PubMed, Scopus, and Web of Science. The search strategy employed a combination of Medical Subject Headings (MeSH) terms and keywords, including (“Antidepressive Agents” [Mesh] OR “Tricyclic Antidepressive Agents” [Mesh] OR “Selective Serotonin Reuptake Inhibitors” [Mesh]) AND (“Stomach Ulcer” [Mesh] OR “Gastric Ulcer” [Mesh]) AND (“Rats” [Mesh] OR “Mice” [Mesh]). The inclusion requirements were experimental studies published in the last five decades, between 1974 and 2024, containing abstracts written in English. Exclusion criteria were: studies published before 1974, clinical studies, including case reports, and case-control studies, articles without abstracts, and articles containing the gastroprotective potential of medicines that are not antidepressants. The selection process is presented in a PRISMA flow diagram (Fig. 1). This systematic review was registered in the PROSPERO database.

The authors collected data from each report and worked independently. For the initial selection, the article’s title and abstract were read first, followed by the full content. The papers were classified according to the subjects addressed in this study, and each topic was evaluated independently by the authors. The literature synthesis in this study was accomplished through a careful examination of a database created by the authors. Before the final analysis, searches were repeated to determine whether new studies were available and might be included in the review. Following the initial selection of papers, the studies were entered into the Mendeley^®^ desktop reference management software to eliminate duplication.

After completing the search strategy, data were extracted based on identification of the article, methodological characteristics of the study, sample number, experimental model, results/conclusion, and assessment of methodological rigor.

The risk of bias assessment was performed using the SYRCLE RoB Tool for Animal Studies (Systematic Review Center for Laboratory Animal Experiments). In brief, seven domains were evaluated for biases, including selection, performance, detection, attrition, reporting, and other sources of bias, which were categorized as “low,” “high,” or “unclear.” When all guiding questions for a domain were “Yes” or “Probably Yes,” the study was rated as low risk. If answers to the domains were unclear, the study was classified as uncertain.

## Results

This review sought experimental studies about antidepressants with beneficial activity against gastric ulcers over the last 50 years. As shown in Fig. 1, the primary search identified 163 articles to guide the discussion of the data. Before the screening, 40 duplicate records were removed. After an initial screening of titles, abstracts, and full text and exclusion of data not about the review of interest, repetitions from other included literature, and publication date before the stipulated date for review, 27 articles were selected. After the manuscript revision 1 further study was included, resulting in 28 articles reviewed. The sample size was limited to studies that met these criteria and were available in English.

Based on the information taken from these publications, 50 independent experimental records were found, taking into account each particular combination of ulcer-inducing agent (such as ethanol, an NSAID, or stress) and study as a separate experimental condition, with the effect of the antidepressant on the ulcerated area or on the gastric ulcer index as the primary outcome. They were counted in several experimental records since ten of the included studies assessed various gastric ulcer models. Every study used rodent models of acute gastric ulcers, with three using mice and twenty-four using rats. The impact on female animals was only assessed in one study. In terms of ulcerogenic agents, the most often utilized model in the examined studies was the stress-induced ulcer model, which was found in 15 experimental records (55.5%), followed by the induction model by NSAID administration, which was found in 12 records (44.4%).

Selective serotonin reuptake inhibitors (SSRIs) were the most commonly studied antidepressants, appearing in 14 studies (51.8%), and tricyclic antidepressants were the second most evaluated class, with 13 studies (48.1%). Seven studies (25.9%) included multiple classes of antidepressants, such as serotonin-norepinephrine reuptake inhibitors and monoamine oxidase inhibitors. The included studies generally showed significant heterogeneity in experimental methods, including variations in antidepressant dosages, delivery schedules, and outcome assessment techniques.

The risk of bias assessment was performed using the SYRCLE Risk of Bias (RoB) Tool for Animal Studies. The results of the analysis according to the domains are shown in the Supplementary Material (Fig. 1SM). Across the included studies, the evaluated domains comprised selection bias, performance bias, detection bias, attrition bias, reporting bias, and other potential sources of bias. Selection bias was frequently rated as unclear, as details regarding random generation and allocation concealment were often not reported. Similarly, performance and detection biases were predominantly classified as unclear due to the lack of information on blinding of investigators, and outcome assessors. Attrition bias was generally low when outcome data were complete, although some studies did not clearly describe exclusions or losses. Reporting bias was difficult to assess in most cases, as protocols were not available for comparison. Overall, most domains were classified as having an “unclear” risk of bias, primarily due to insufficient reporting of methodological details. Only a limited number of studies provided adequate information to be judged as having a low risk of bias in specific domains. Therefore, the findings indicate a moderate to unclear risk of bias among the included studies, highlighting the need for improved methodological transparency and reporting in future animal research.

## Discussion

Evaluating these studies made it possible to identify that several antidepressants can protect the gastric mucosa against different ulcerogenic agents used in acute ulcer models, including ethanol, reserpine, indomethacin, stress, and pylorus ligation. These findings are discussed below, separated into topics according to the antidepressant class.

### Evidence for the gastroprotective impact of tricyclic antidepressants

Tricyclic antidepressants (TCAs) are a type of medication used to treat depression, particularly in refractory cases. Its main structural feature is the presence of three carbon rings forming an imino dibenzyl nucleus, and its mechanism of action involves the non-selective inhibition of serotonin (5-HT) and norepinephrine (NE) reuptake, inhibiting serotonin transporters (SERT) and noradrenaline (NET), respectively, increasing the availability of these substances in the synaptic cleft (Stahl 2014).

According to Moraczewski (2023), one of the major limitations of the use of tricyclics, which are currently used as a second-line treatment for depression, is the significant set of adverse effects caused by their action on multiple receptors, including histamine (H1), α1-adrenergic, and muscarinic cholinergic (M1). These effects can cause weight gain, drowsiness, constipation, visual blurring, and dry mouth. TCAs are used to treat neuropathic pain, obsessive-compulsive disorder (OCD), and nocturnal enuresis, in addition to their gastroprotective activity, which reduces the formation of gastric ulcers (Xu, 2017; Schneider, 2019).

Table 1 summarizes the experimental data for the gastroprotective action of TCAs, which will be examined more below:

**Table 1:** Experimental evidence for tricyclic antidepressants’ antiulcer potential

ANTIDEPRESSANT (dose, administration route)	Ulcerogenic agent	Animal	Main findings	References
Amitriptyline (10 mg/kg, i.p)	Reserpine	Male rats	The administration of amitriptyline (10 mg/kg, i.p) had a protective effect, reducing the number of ulcers in each stomach and the severity of the lesions; however, it did not induce the full extinction of ulcers in the gastric mucosa	Sandor and Cuparencu, 1977
Trimipramine (5 mg/kg/h, i.v.)	Ethanol	Female rats	Trimipramine (5 mg/kg/h, i.v.) administration caused changes in the ionic flow, with an increase in the depletion of H^+^ ions and a decrease in the flows of Na + and K+ ions, as well as the potential difference of the gastric mucosa, resulting in a reduction in the score lesions when compared to the control group, demonstrating that it is beneficial in the treatment of gastric ulcers	Deregnaucourt 1979
Desipramine (5 and 10 mg/kg, s.c)	hypothalamic injury	Male rats	The treatment of desipramine (5 and 10 mg/kg, s.c.) reduced the extent of gastric lesions, which the authors attributed to catecholamines’ protective role in against acute ulcers	Nobrega and Wiener 1983
Trimipramine (5 mg/kg, i.p)	Stress Indomethacin Aspirin Pyloric ligation Reserpine	Male rats	Trimipramine (5 mg/kg, i.p.) reduced ulcer rate in all experimental models while also reducing lesion size, gastric secretion volume, total acidity, and free acidity secretion in the ligation pyloric model	Aguwa and Ramanujam 1984
Demethylimipramine (1 mL/kg, i.v and 0.1 mL/kg, i.c) Doxepin (1 mL/kg, i.v and 0.1 ml/kg, i.c)	Pyloric ligation	Male rats	Both drugs administered at doses of 1 ml/kg (i.v) and 0.1 ml/kg (i.c), reduced stomach output significantly, with the effect being stronger when administered intracerebrally rather than intravenously	Pendleton et al. 1984
Imipramine, (0.2, 2 and 5 mg/kg, i.p and 0.5 mg/kg e 1 µg/rato, i.c)	Stress Pyloric ligation	Male rats	In the stress model, imipramine (0.2, 2, and 5 mg/kg, i.p and 0.5 and 1 µg/rat i.c) reduced the incidence and severity of stomach lesions. It was more efficacious when delivered intraperitoneally compared to the intracisternal route In the pyloric ligation model, imipramine at a dose of 5 mg/kg reduced stomach volume, increased pH, and suppressed acid output, indicating that this drug has an antiulcerogenic effect	Hernandez and Xue 1989
Imipramine (0,05, 0,5 and 5 mg/kg, i.p)	Thyrotropin-releasing hormone (THR)	Male rats	Imipramine (0.05, 0.5, and 5 mg/kg, i.p.) reduced the occurrence and severity of lesions caused by TRH intracisternal administration, as well as decreased acid secretion in response to TRH intracisternal application	Hernandez et al. 1990
Dothiepin (25, 50 and 100 mg/kg, p.o)	Ethanol Aspirin Indomethacin Pyloric ligation Stress	Male rats	Dothiepin (25, 50, and 100 mg/kg, p.o.) inhibited the formation of stomach lesions in ulcer models produced by ethanol, aspirin, indomethacin, and stress In the pyloric ligation model, dothiepin administration (25 and 100 mg/kg, p.o.) reduced the overall acidity and protein content of gastric juice while leaving stomach volume constant	Sen et al. 2000
Amitriptyline (15, 40 and 75 mg/kg, p.o)	Ethanol Aspirin Indomethacin Pyloric ligation Stress	Male rats	Amitriptyline (15, 40, and 75 mg/kg, p.o.) reduced the size of lesions in ethanol-induced ulcers In aspirin-induced ulcers, those given the medication demonstrated a decrease in ulcerative lesions In the indomethacin-induced ulcer model, amitriptyline effectively inhibited ulcer formation. Furthermore, stress-induced ulcerative damage was reduced in groups treated with the medication Pre-treatment with amitriptyline in rats subjected to pyloric ligation reduced the total acidity of proteins included in the gastric juice; however, a significant reduction in the number of gastric contents was found only at a dose of 75 mg/kg (p.o.)	Sen et al. 2000
Opipramol (25, 50 and 100 mg/kg, p.o)	Indomethacin	Male rats	Pretreatment with opipramol had antiulcerogenic effects at all doses (25, 50, and 100 mg/kg, p.o.), compared to the control group. Furthermore, there was an increase in the levels of antioxidants such as nitric oxide (NO), glutathione (GSH), and the enzyme superoxide dismutase (SOD), a decrease in the enzyme catalase (CAT), and a decrease in the levels of oxidative stress-related, compounds such as malondialdehyde (MDA) and myeloperoxidase (MPO)	Dursun et al. 2009a, b
Amitriptyline (10 e 20 mg/kg, i.p)	Stress Indomethacin Reserpine	Male rats	Pre-treatment with amitriptyline (10 and 20 mg/kg, i.p) in the stress-induced ulcer model reduced the number of lesions and intraluminal bleeding; however, there was no improvement when provided at a dose of 5 mg/kg. kg, i.p. Also, pre-treatment with amitriptyline (10 and 20 mg/kg i.p.) reduced indomethacin-induced ulcers In the reserpine-induced ulcer model, pretreatment with amitriptyline (5, 10, and 20 mg/kg i.p) reduced lesions and intraluminal bleeding in all rats tested	Ji et al. 2012
Amitriptyline (10 mg/kg, i.p)	Stress Indomethacin	Male rats	Pre-treatment with amitriptyline (10 mg/kg, i.p) lowered the levels of dual oxidase 2 (DUOX2), NADPH oxidase 2 (NOX2), and NADPH oxidase 4 (NOX4). Amitriptyline (10 mg/kg i.p) reduced the levels of DUOX2, NADPH oxidase 1 (NOX1), and NOX4	Cao et al. 2020
Amitriptyline (20 mg/kg, i.p)	Stress	Male rats	Pre-treatment with amitriptyline (10 mg/kg, i.p) enhanced ASPA enzyme levels, which was associated with less stomach lesions	Yao et al. 2022

Sandor and Cuparencu, 1977 initiated such research by testing the effect of amitriptyline (10 mg/kg, i.p.) on male rats with reserpine-induced ulcers. The trial showed that amitriptyline had a gastroprotective effect, reducing the number of ulcers in each stomach and their severity. Despite discovering that the antidepressant has a gastroprotective effect, the study was unable to establish an underlying mechanism of action for the gastroprotective effect of amitriptyline.

Deregnaucourt (1979) investigated the potential effects of trimipramine on a gastric ulcer model caused by ethanol and hydrochloric acid (HCl) in female rats. It is recognized that ethanol can breach the gastric mucosa’s protective barrier by inducing changes in the mucus and cell structure. After the induction of the lesions, trimipramine (5 mg/kg/h, i.v.) was administered and changes in the ionic flux were observed, with an increase in the depletion of H^+^ ions and a reduction in the fluxes of Na^+^ and K^+^, as well as the difference in potential of the gastric mucosal membranes, with a reduced lesion score when compared to the control group, suggesting the beneficial action of trimipramine on ethanol-induced stomach ulcers in female rats.

Despite the finding of trimipramine’s efficacy against stomach ulcers, Deregnaucourt (1979) recognized limits in understanding the drug’s gastroprotective mechanisms. Furthermore, based on another study using a molecule with structural chemical similarities to trimipramine, the author proposed that trimipramine’s mode of action may include antagonism in the H2 receptor.

Following the same research purpose, Nobrega and Wiener (1983) investigated the impact of medications that affect noradrenergic pathways on ulcers generated by hypothalamic damage in male rats. The authors discovered that desipramine (5 and 10 mg/kg, s.c) reduced the gastric lesions. However, the study did not clarify the mechanisms of action underpinning such outcomes.

Nonetheless, Aguwa and Ramanujam (1984) continued their investigation into the antiulcerogenic effects of trimipramine in male rats whose ulcers were caused by stress, indomethacin, aspirin, reserpine, and pyloric ligation. Trimipramine (5 mg/kg, i.p.) reduced the ulcer rate in all experimental models, as well as the size of the lesions, volume of gastric secretion, total acidity, and free acid secretion in the pyloric ligation model (Aguwa and Ramananujam, 1984). Given that the dose administered was capable of reversing the damage caused by different models of ulcer induction while not promoting side effects such as mydriasis, the authors suggest that the antiulcerogenic effect of trimipramine should not be related to its anticholinergic action, but rather to another mechanism, yet to be clarified, capable of increasing the resistance of the gastrointestinal mucous layer.

Pendleton et al. (1984) conducted a comparative study between the antisecretory effects of desmethyl imipramine (1 mL/kg, i.v and 0.1 ml/kg, i.c.) and doxepin (1 mL/kg, i.v and 0.1 mL/kg, i.c.), discovering that both drugs administered at doses of 1 ml/kg (i.v) and 0.1 mL/kg (i.c.) reduced gastric secretion, and this effect was more powerful when the drugs were administered intracerebral than intravenously, indicating central effects media.

Hernandez and Xue (1989) researched the gastroprotective activity of TCAs, examining the role of imipramine in preventing stress-induced ulcers and pyloric ligation in male rats. In the stress model, imipramine at dosages of 0.2, 2, and 5 mg/kg (i.p.) and 1 µg/rat (i.c.) reduced the incidence and severity of stomach lesions, with intraperitoneal administration being more efficient than the intracisternal route. It is worth noting that these findings contradicted the effects discovered by Pendleton et al. (1984) for desmethyl imipramine regarding the impact of the route of administration on the gastroprotective effect. In comparison to the pyloric ligation model, administering imipramine (5 mg/kg, i.p) reduced the gastric volume and acidity, and increased pH, indicating that this medicine has an antiulcerogenic and anti-gastric acid secretion effect (Hernandez and Xue 1989).

Hernandez et al. (1990) studied the antiulcer effects of imipramine in male rats with and without pyloric ligation. Thyrotropin-releasing factor (TRH, 1 µg/rat, i.c) stimulates neuronal cholinergic activity, increasing gastric secretion and ulcer formation (Taché, 1989). The authors found that administering imipramine (0.05, 0.5, and 5 mg/kg, i.p) reduced the incidence and severity of TRH-induced gastric lesions, decreasing acid secretion in male rats with pyloric ligation. These data suggested that the activity of imipramine may be associated with the suppression of stomach acid secretion, especially under parasympathetic stimulation.

Sen et al. (2000) investigated the effects of dothiepin on stomach ulcers caused by stress, ethanol, aspirin, indomethacin, and pyloric ligation in male rats. The authors reported that dothiepin (25, 50, and 100 mg/kg, p.o.) prevented the formation of stomach lesions in ulcer models caused by ethanol, aspirin, indomethacin, and stress. The authors discovered that administering dothiepin (25 and 100 mg/kg, p.o.) reduced the acidity and protein content of the gastric juice without significantly affecting the pH or stomach volume.

Following the inquiry into the gastroprotective function of TCAs, Sen et al. (2002) conducted a new study to assess the effects of amitriptyline on stomach ulcers produced by various models in male rats. Thus, oral treatment of amitriptyline (15, 40, and 75 mg/kg) reduced the size of ulcers caused by ethanol, aspirin, and indomethacin. Furthermore, stress-induced ulcerative lesions were significantly reduced in groups pre-treated with the medication. The study also found that pre-treatment with amitriptyline in rats before pyloric ligation reduced overall acidity and protein levels in gastric juice. Sen et al. (2002) found that amitriptyline outperformed dothiepin in terms of gastroprotection in models of pyloric ligation, indomethacin, alcohol, and stress.

Based on the realization that stress and continuous use of NSAIDs and alcohol promote an increase in the synthesis of reactive oxygen species (ROS), which play an important role in the formation of ulcerative lesions in the gastrointestinal tract. Bhattacharyya et al. (2014); Dursun et al. (2009a, b) investigated whether the gastroprotective effects found in TCAs may also be attributed to antioxidant action. The authors conclude that the activation of enzymatic and non-enzymatic antioxidant defenses, together with the inhibition of oxidant mechanisms harmful to gastric tissue play an important role in the action of opipramol’s antiulcer effect. This finding, therefore, should broaden the choices for the clinical application of this drug.

Ji et al. (2012) sought to compare the effects of four antidepressants from different classes (duloxetine, amitriptyline, fluoxetine, and mirtazapine) in male rats subjected to different ulcer induction models involving stress, indomethacin, and reserpine, based on the understanding that gastric ulcers have a multifactorial etiology and taking into account the numerous studies on the application of antidepressants in the treatment of this condition.

Still, in the study by Ji et al. (2012), pre-treatment with amitriptyline (10 and 20 mg/kg, i.p) in the stress-induced ulcer model reduced the extent of lesions and intraluminal bleeding, and there was no significant improvement in the above parameters when administered at a dose of 5 mg/kg (i.p). Regarding indomethacin induction, pre-treatment with amitriptyline (10 and 20 mg/kg, i.p) improved both the number of lesions and intraluminal bleeding, with no significant improvement at a dose of 5 mg/kg (i.p). Finally, Ji et al. (2012) reported that in the reserpine-induced ulcer model, pre-treatment with amitriptyline (5, 10, and 20 mg/kg, i.p) reduced lesions and intraluminal bleeding in all animals tested. Given the data obtained, the authors conclude that amitriptyline provided gastroprotective effects primarily through its action on neural pathways involved in cholinergic and vagal tone.

In their search for mechanisms of action, Cao et al. (2020) attempted to determine the role of antidepressants in modifying the levels of NADPH oxidases (NOX), enzymes responsible for ROS formation. In the stress-induced model, amitriptyline (10 mg/kg, i.p) pretreatment reduced the levels of dual oxidase 2 (DUOX2), NADPH oxidase 2 (NOX2), and NADPH oxidase 4 (NOX4). Also, amitriptyline (10 mg/kg, i.p) reduced DUOX2, NADPH oxidase 1 (NOX1), and NOX4 levels in the indomethacin-induced ulcers. The researchers were able to deduce a peripheral molecular target for antidepressants that alter monoamines, such as TCAs, which may it would be beneficial to learn more about the mechanics of this sort of medicine beyond monoamine control.

Furthermore, Yao et al. (2022) aimed to clarify the molecular processes behind TCAs’ gastroprotective action; male rats were exposed to the stress-induced ulcer paradigm. Pre-treatment with amitriptyline (10 mg/kg, i.p) was shown to increase the levels of the enzyme aspartoacylase (ASPA) present in oligodendrocytes, which is responsible for the hydrolysis of N-acetyl-aspartate (NAA), a neurometabolic produced in the mitochondria of brain tissue, which functions as a marker of neuronal vitality and is elevated when the organism is subjected to stressful situations. High levels of ASPA were associated with lower NAA levels and stomach lesions, implying that this is one of the molecular processes underlying antidepressant gastroprotection.

**Fig. 2:**
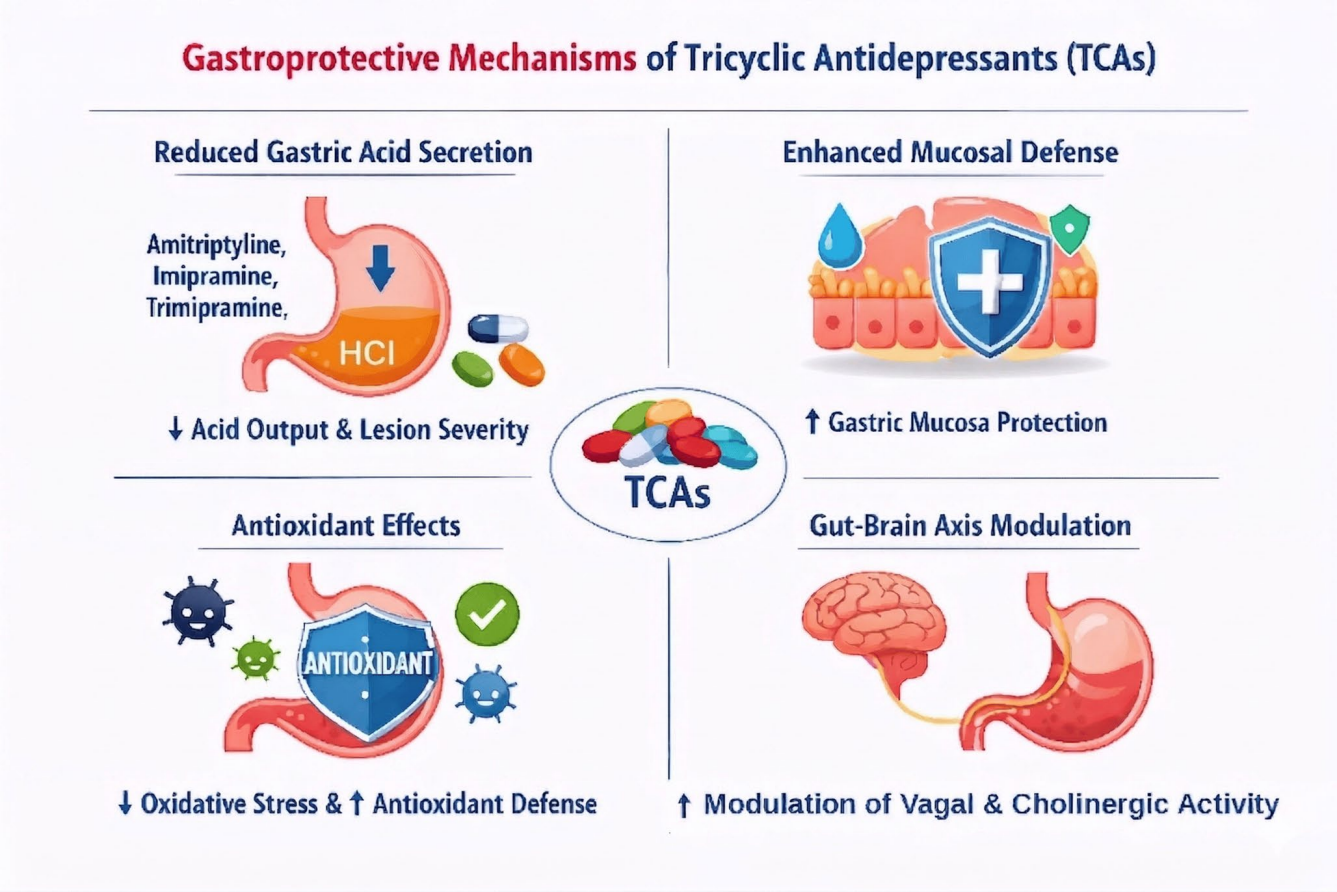
Gastroprotective mechanisms of tricyclic antidepressants (TCAs). TCAs exert gastroprotective effects through multiple mechanisms: reducing gastric acid secretion, enhancing mucosal defense, exhibiting antioxidant properties, and modulating the gut-brain axis, especially through vagal and cholinergic tone

The gastroprotective effects of tricyclic antidepressants (TCAs) can be attributed to several mechanisms, aligning with established theories of ulcerogenesis as shown in Fig. 2. Firstly, TCAs have been shown to reduce gastric acid secretion, a key factor in ulcer formation, as demonstrated by studies where trimipramine, amitriptyline, and imipramine decreased acid output and lesion severity (Aguwa and Ramanujam 1984; Hernandez and Xue 1989; Sen et al. 2000, 2002). Secondly, TCAs may enhance mucosal defense mechanisms, increasing the resistance of the gastrointestinal mucosa to injury (Aguwa and Ramanujam 1984). Thirdly, the antioxidant properties of TCAs contribute to their gastroprotective effects, as evidenced by reduced oxidative stress and enhanced antioxidant defenses in gastric tissue (Bhattacharyya et al. 2014; Dursun et al. 2009a, b; Cao et al. 2020). Lastly, TCAs modulate the gut-brain axis, influencing central mechanisms involved in gastric secretion and mucosal protection, such as vagal and cholinergic tone (Ji et al. 2012; Pendleton et al. 1984). These multifaceted actions underscore the potential of TCAs in preventing and treating peptic ulcers.

### Selective serotonin reuptake inhibitors’ gastroprotective benefits

Selective serotonin reuptake inhibitors (SSRIs) were first used in the clinic in the late 1980s. This class’s mode of action involves inhibiting 5-HT transporters (SERT), which prevents serotonin reuptake by pre-synaptic neurons, increasing its availability in the synaptic cleft and boosting the alleviation of depressive symptoms after some period of usage (Lochmann, 2019). Furthermore, it has been used to treat a variety of diseases, including generalized anxiety disorder (GAD), obsessive-compulsive disorder (OCD), and post-traumatic stress disorder (PTSD) (Stahl 2014). More recently, studies have been conducted to investigate the gastroprotective potential of SSRIs, as shown in Table 2 and described further below.

**Table 2:** Experimental evidence for selective serotonin reuptake inhibitors antidepressants’ antiulcer potential

Antidepressant (dose, administration route)	Ulcerogenic agent	Animal	Main findings	References
Fluvoxamine (25, 50, 100 and 200 mg/kg, p.o)	Indomethacin	Male rats	Fluvoxamine (25, 50, 100, and 200 mg/kg, p.o.) inhibited ulcer formation, increasing GSH and NO and decreasing MDA and MPO	Dursun et al. 2009a, b
Paroxetine (1–10 mg/kg, p.o)	Indomethacin	Male rats	When compared to the group that received only indomethacin, pre-treatment with paroxetine (1–10 mg/kg p.o.) worsened gastric lesions	Takeuchi et al. 2011
Citalopram (5, 10 and 20 mg/kg, p.o)	Stress Pyloric ligation	Male rats	In stress-induced ulcers, pre-treatment with citalopram (5, 10, and 20 mg/kg p.o.) in a single dose had no effect, whereas 14-day pre-treatment promoted gastroprotection.	Saxena and Singh 2011
Paroxetine (20 mg/kg, p.o)	Stress	Male mice	Pre-treatment with paroxetine for 8 and 22 consecutive days reduced ulcerated areas but did not affect serum corticosterone levels	Takahashi et al., 2012
Fluoxetine (5 and 10 mg/kg, i.p)	Stress Indomethacin Reserpine	Male rats	In the stress-induced animal, fluoxetine (5 and 10 mg/kg, i.p) exhibited gastroprotective properties. In ulcers caused by indomethacin, fluoxetine administration at 10 and 20 mg/kg (i.p.) promoted gastroprotection. In reserpine-induced ulcers, fluoxetine treatment at 10 mg/kg (i.p.) also demonstrated an antiulcerogenic effect	Ji et al. 2012
Fluoxetine (10 mg/kg, i.p)	Acute cold containment stress	Male and female rats	Male rats responded better to fluoxetine (10 mg/kg i.p.) than female rats. In stressed male rats, gastric MDA increased while gastric CAT, NO, and cerebral GABA decreased.	Abdel-Sater et al. 2012
Fluoxetine (20 mg/kg, i.p)	Ethanol Indomethacin	Male rats	Pre-treatment with fluoxetine (20 mg/kg, i.p) in both models prevented stomach ulcers regulating gastric serotonin and histamine levels, COX-1 and 2 enzymes, NO, GSH, MPO, TNF, and gastric pH	Sokar et al. 2016
Fluvoxamine (50 mg/kg, p.o)	Stress	Male rats	Pre-treatment with fluvoxamine (50 mg/kg, p.o.) decreased gastrointestinal lesions promoting gastric mucosa and tissue regeneration and reducing oxidative stress	Elsaed et al., 2018
Fluvoxamine (50 and 100 mg/kg, p.o)	Indomethacin Stress	Male mice	Fluvoxamine (50 and 100 mg/kg, p.o.) reduced ulcers caused by indomethacin and stress, and it was discovered that both indomethacin and stress might raise Hsp70 expression, with fluvoxamine pre-treatment increasing it even more	Khotib et al. 2019
Fluoxetine (10 mg/kg, i.p)	Stress Indomethacin	Male rats	Fluoxetine (10 mg/kg i.p.) decreased NOX2 and DUOX2 levels in the stress-induced model while increasing NOX1 and DUOX2 levels in the indomethacin-induced ulcer model	Cao et al. 2020
Fluvoxamine (50, 100 mg/kg, i.p and 18.6 µg i.c.v)	Stress	Male mice	Fluvoxamine (50 and 100 mg/kg i.p and 8.6 µg i.c.v) reduced ulcerative lesions, however only the intraperitoneal 100 mg/kg dose decreased intraluminal bleeding. Furthermore, intraperitoneal injection produced more effective effects. The combination of fluvoxamine and ondansetron had no greater antiulcerogenic benefit over fluvoxamine monotherapy (100 mg/kg, p.o)	Rahmadi et al. 2021
Fluvoxamine (25 and 50 mg/kg, v.o) Mirtazapine (15 and 30 mg/kg, p.o)	Stress Pyloric ligation	Male rats	In the stress-induced model, treatment of fluvoxamine (25 and 50 mg/kg, p.o.) and mirtazapine (15 and 30 mg/kg, p.o.) reduced the number of lesions, with mirtazapine (30 mg/kg, p.o. kg) having a greater impact over fluvoxamine dosages. Both drugs inhibited ulcer formation while restoring PGE2 levels and increasing antioxidant concentrations in the pylorus-ligated model	Abdel-Hamed et al. 2022
Fluoxetine (10 mg/kg i.p)	Stress	Male rats	Pre-treatment with fluoxetine (10 mg/kg i.p.) increased ASPA enzyme levels and it was associated with a decrease in stomach lesions	Yao et al. 2022

Dursun et al. (2009a, b) studied the antiulcerogenic and antioxidant capabilities of fluvoxamine in male rats with indomethacin-induced ulcers, based on the fact that NSAIDs are intimately associated with gastric mucosa damage caused by prostaglandin depletion (Bindu et al. 2020). Fluvoxamine (25, 50, 100, and 200 mg/kg, p.o.) was found to be effective in inhibiting ulcer formation, as well as increasing antioxidant levels such as GSH and NO and lowering concentrations of oxidative stress species such as MDA and MPO, implying that their gastroprotective role is associated with the reduction of oxidative mechanisms and subsequent activation of antioxidant mechanisms.

Previous research has shown that combining SSRIs and NSAIDs may increase the risk of gastrointestinal bleeding due to impaired platelet function (Weinrieb et al., 2005). Despite the fluvoxamine data, Takeuchi et al. (2011) investigated the effects of paroxetine treatment on male rats with indomethacin-induced ulcers. Contrary to the findings of Dursun et al. (2009a, b), the authors confirmed that pre-treatment with paroxetine (1–10 mg/kg, p.o.) worsened stomach lesions as compared to the group that only received indomethacin, resulting in the formation of hemorrhagic lesions. To understand the various mechanisms involved in the drug’s activity, the authors gave exogenous 5-HT and observed the formation of lesions identical to those generated by the action of paroxetine. Based on this observation, we wanted to determine which 5-HT receptors were associated with the worsening effect of paroxetine.

Thus, after the administration of several 5-HT receptor antagonists, Takeuchi et al. (2011) found that ondansetron (5-HT3 receptor antagonist) was the only drug capable of reversing the damage to the gastric mucosa caused by paroxetine, indicating therefore, the route of action involved in the pathogenesis of this SSRI is due to the activation of 5HT-3 receptors. Furthermore, antisecretory drugs such as omeprazole and atropine, as well as mucosal protectors such as rebamipide and irsogladine, were used to promote the inhibition of paroxetine-induced damage, implying that gastric acid-mediated damage is one of the mechanisms responsible for injury worsening. In addition, highlighting the need for caution in the administration of NSAIDs and paroxetine, Yamaguchi et al. (2008) described that the coadministration of low-dose aspirin and paroxetine significantly enhanced the gastric fluid secretion and acid output, resulting in gastric bleeding.

Takahashi et al. (2012) investigated the effects of consecutive administration of paroxetine in male mice with stress-induced ulcers after previous research demonstrated that antidepressants can enhance the process of neurogenesis in the hippocampal region, providing some adaptation to stress. In contrast to the results of Takeuchi et al. (2011), the study of Takahashi et al. (2012) showed that the pretreatment with paroxetine (20 mg/kg, p.o.) for 8 and 22 consecutive days decreased ulcerated stomach mucosa. The scientists hypothesized that the drug’s protective impact on the stomach mucosa was caused by the production of a state of resilience and adaptability to stress, as they also performed behavioral tests on these animals, rather than factors connected to the gastric tissue. Therefore, comparing the results of both studies is possible to observe that the gastroprotective effects of paroxetine depends of the ulcers’ etiologic factors, probably making this drug useful in conditions related to social stress but not in patients under treatment with an NSAID.

Saxena and Singh (2011) evaluated the antiulcerogenic effects of citalopram in male rats with stress-induced ulcers and pyloric ligation. In stress-induced ulcers, pre-treatment with citalopram (5, 10, and 20 mg/kg, p.o.) in a single dose did not demonstrate benefits. However, the pre-treatment carried out for 14 consecutive days showed a gastroprotective effect, reducing levels of plasma corticosterone, a hormone linked to stress, as well as nitrite levels and lipid peroxidation, which are related to oxidative stress, in addition to increasing levels of prostaglandin E_2_ (PGE_2_) is associated to the suppression of gastric secretion. In this study, hexosamine levels increased (connected to gastric mucus secretion) and microvascular permeability decreased only during the 14-day pre-treatment with citalopram at an oral dose of 10 mg/kg.

Furthermore, Saxena and Singh (2011) aimed to identify the various pathways involved in citalopram’s antiulcerogenic activity. Knowing that prostaglandins restrict stomach secretion by activating ATP-sensitive potassium channels, the study aimed to determine whether citalopram’s gastroprotective effects required the activation of these same channels. Pre-treatment with glibenclamide, an ATP-sensitive potassium channel blocker, prevented citalopram’s gastroprotective properties. The gastroprotective effect of citalopram was also reduced in animals treated with N-ethylmaleimide (NEM) or L-(G)-nitro-L arginine-methyl-ester (L-NAME), a chelator of non-protein sulfhydryl groups (SH-NP) and an inhibitor of the enzyme nitric oxide synthase, respectively, indicating the importance of such pathways in the observed effect. Ulcers caused by the following pyloric ligation, 14 days of pre-treatment with citalopram (5, 10, and 20 mg/kg p.o.) had no significant gastroprotective effect. Thus, the authors propose that citalopram is capable of interfering with multiple processes connected to gastric ulcerogenesis, but does not interfere with acid secretion, making it a drug with potential for treating stress-induced ulcers (Table 3).

**Table 3:** Experimental evidence of the antiulcer potential of antidepressants from other classes

Antidepressant(dose, administration route)	Ulcerogenic agent	Animal	Main findings	References
Mirtazapine (15, 30, and 60 mg/kg, p.o)	Indomethacin	Male rats	Oral Mirtazapine reduced the development of gastric lesions and hyperemia in a dose-dependent manner	Bilici et al. 2008
Tianeptine (6, 12, and 25 mg/kg, p.o) Trazodone (12, 25, and 50 mg/kg, p.o) Venlafaxine (12, 25, and 50 mg/kg, p.o)	Indomethacin	Male rats	Trazodone and Venlafaxine, orally, did not prevent stomach mucosal lesions, however, tianeptine (6, 12, and 25 mg/kg p.o.) did. Tianeptine treatment increased enzymatic and non-enzymatic antioxidant defenses in the stomach mucosa while decreasing oxidative stress indicators	Suleyman et al. 2009
Duloxetine (5, 10 and 20 mg/kg, i.p) Mirtazapine (5, 10 and 20 mg/kg, i.p)	Stress Indomethacin Reserpine	Male rats	In the stress-induced model, duloxetine (5, 10, and 20 mg/kg, i.p.) and mirtazapine (10 and 20 mg/kg, i.p.) promoted gastroprotective. Duloxetine (20 mg/kg i.p) and mirtazapine (5, 10, and 20 mg/kg, i.p) also promoted gastroprotection in indomethacin-included ulcers. In the case of reserpine-induced ulcers, duloxetine (5, 10, and 20 mg/kg i.p) and mirtazapine (5 and 10 mg/kg) were antiulcerogenic	Ji et al. 2012
Moclobemide (10, 20, 40, 80, and150 mg/kg, p.o)	Indomethacin	Male rats	Moclobemide at all doses reduced ulcerative lesions, and the drug increased antioxidant levels while decreasing oxidative stress levels	Albayrak et al. 2014
Duloxetine (5 and 20 mg/kg, i.p) Moclobemide (10, 30 and 100 mg/kg, i.p	Stress Indomethacin	Male rats	Duloxetine reduced bleeding points in the stomach mucosa in a dose-dependent manner, but only the 20 mg/kg dose was effective in lowering MDA levels. Furthermore, it was found that the same dose effectively inhibited the rise in NADPH oxidase activity Duloxetine treatment reduced the expression of NOX1, NOX2, DUOX2, and NOX4	Cao et al. 2020
Mirtazapine (15 e 30 mg/kg, p.o)	Stress Pyloric ligation	Male rats	Mirtazapine (15 and 30 mg/kg, p.o.) reduced the number of gastric lesions while increasing PGE2 levels and normalizing oxidative indicators. Mirtazapine treatment lowered stomach volume and total and free acidity	Abdel-Sater et al. 2012
Duloxetine (5 and 20 mg/kg, i.p) Moclobemide (20 mg/kg, i.p)	Stress	Male rats	Pre-treatment with duloxetine or moclobemide increased ASPA enzyme levels, which was associated with less stomach lesions	Yao et al. 2022

Based on the notion that biological sex has a distinct influence on drug response, Abdel-Sater et al. (2012) studied the variations in the gastroprotective activity of fluoxetine in male and female rats with stress-induced ulcers. It was discovered that when exposed to stress, 100% of the male sample and 80% of the female sample developed gastric ulcers, indicating that the male biological sex is more prone to acquiring gastric lesions in response to stress. Treatment with fluoxetine (10 mg/kg, i.p) decreased lesions and stomach acidity while improving gastric capacity. These effects were more substantial in the male sample than in the female sample, indicating that men are more receptive to the gastroprotective potential of SSRI. Furthermore, the study assessed gamma-aminobutyric acid (GABA) levels, as the GABAergic system is associated with stress response modulation, and found that fluoxetine treatment increased cortical GABA levels in both sexes, with no significant difference.

Abdel-Sater et al. (2012) proposed that the different responses to fluoxetine presented by both biological sexes may occur due to issues related to the pharmacokinetics and metabolism of fluoxetine, as well as hormonal differences related to estrogen, which stimulates the production of calcitonin gene-related-peptide (CGRP), which is released by sensory neurons, attenuating stress-induced gastric injuries, which corroborates the findings listed above that females are less likely to develop gastric ulcers as a result of stress than males.

Based on the understanding that gastric ulcers have a multifactorial etiology, and taking into account the numerous studies on the use of antidepressants in the treatment of this condition, Ji et al. (2012) compared the effects of four antidepressants from different classes (duloxetine, amitriptyline, fluoxetine, and mirtazapine) in male rats subjected to various gastric ulcer models. In the stress-induced animal, fluoxetine (5 and 10 mg/kg, i.p) showed an effective gastroprotective effect. In ulcers caused by indomethacin, fluoxetine (10 and 20 mg/kg, i.p.) improved gastroprotection and reduced intraluminal bleeding. In reserpine-induced ulcers, fluoxetine (10 mg/kg, i.p) had an antiulcerogenic effect while reducing intraluminal bleeding.

Ji et al. (2012) proposed that increased serotonin availability is one of the mechanisms responsible for fluoxetine’s gastroprotective effect, citing previous studies that found that intracerebroventricular injections of serotonin and noradrenaline in the central nucleus of the amygdala were capable of attenuating the injuries produced by stress-induced ulcers, indicating that the event was mediated primarily by the CNS. Furthermore, another putative mechanism involved is prostaglandin and mucus formation, indicating a local influence on stomach tissue, particularly in light of the NSAID induction model results.

Sokar, Elsayad, and Ali (2016) investigated the mechanisms underlying fluoxetine’s gastroprotective effect in male rats with ulcers caused by alcohol and indomethacin. The results showed that pre-treatment with fluoxetine (20 mg/kg, i.p) in both induction models reduced the development of ulcers in the stomach mucosa by stabilizing gastric serotonin and histamine levels, as well as the enzymes cyclooxygenases 1 to baseline levels. (COX1) and 2 (COX2), NO, stomach pH, and GSH, but MPO and tumor necrosis factor (TNF) levels were reduced. As a result, the authors believe that fluoxetine’s gastroprotective action is due to several pathways, and they underline that the drug should be used as an effective therapy option for gastrointestinal injuries.

Elsaed et al. (2018) sought to evaluate the gastroprotective and antioxidative effects of fluvoxamine in male rats with stress-induced ulcers, based on the principle that the pathogenesis of gastric ulcers is related to a biochemical imbalance that results in excess production of free radicals, as well as knowledge of the antiulcerogenic properties of SSRIs. Fluvoxamine pre-treatment (50 mg/kg, p.o.) reduced the number and size of gastric lesions while also increasing stomach mucosal and glandular tissue regeneration. In terms of biochemical parameters, the drug increased the levels of antioxidant substances such as GSH, SOD, and CAT while decreasing the concentration of oxidative stress-related species such as lipoperoxidase (LPO), implying that its gastroprotective role is associated with activation of antioxidant pathways.

Khotib et al. (2019) investigated the potential link between fluvoxamine’s gastroprotective effects and the overexpression of the heat shock protein Hsp70, a molecule with gastroprotective activity that increases in cases of aggression to gastric tissue, in male mice with ulcers caused by stress and indomethacin. In the indomethacin-induced model, fluvoxamine (50 and 100 mg/kg, p.o.) was efficient in lowering the extent of the lesions, particularly at a dose of 100 mg/kg, which also reduced intraluminal bleeding. In the stress-induced model, both doses had an antiulcerogenic effect and decreased intraluminal hemorrhage. Khotib et al. (2019) also showed that both indomethacin and stress were able to elevate the expression of 70 kD heat shock protein (Hsp70). Pre-treatment with fluvoxamine was able to further increase its expression.

Cao et al. (2020) investigated the link between several antidepressants, including fluoxetine, and the modification of NADPH oxidase enzymes implicated in oxidative damage induced by stomach ulcers in male rats with ulcers caused by stress and indomethacin, respectively. The scientists found that after administering fluoxetine (10 mg/kg, i.p), NOX2 and DUOX2 levels decreased in the stress induction model, while NOX1 and DUOX2 levels decreased in the indomethacin model. Thus, the authors proposed that the drug’s serotonin modulation is linked to NOX2 expression and that this must be the mechanism underlying the gastroprotection. Furthermore, the authors propose that NOX2 be the focus of investigations targeted at safeguarding the stomach mucosa.

Continuing, Rahmadi et al. (2021) investigated possible differences in the gastroprotective effects of fluvoxamine when injected directly into the CNS versus intraperitoneal administration, as well as the potential benefits of its combination with ondansetron for the treatment of gastric ulcers in mice. Fluvoxamine (50 and 100 mg/kg, i.p. and 8.6 µg, i.c.v) reduced ulcerative lesions in male rats. However, intraperitoneal administration proved more effective.

Furthermore, Rahmadi et al. (2021) investigated the effect of combining fluvoxamine (100 mg/kg) given orally with ondansetron (3 mg/kg), because the latter acts by blocking 5HT3 receptors, and discovered that, while the gastric mucosa protects, there was no superiority in the antiulcerogenic effect of the combination of drugs over monotherapy with fluvoxamine (100 mg/kg, p.o.). Regarding fluvoxamine’s efficacy in preventing stomach ulcers, the study did not define the mechanism involved in the gastroprotection process, implying that more research should be conducted to discover an explanation.

Still regarding fluvoxamine, Abdel-Hamed et al. (2022) compared its antiulcerogenic effects with mirtazapine, as well as the relationship between their antisecretory and antioxidative effects in male rats with stress-induced ulcers and pyloric ligation. In the stress-induced model, the administration of fluvoxamine (25 and 50 mg/kg, p.o.) and mirtazapine (15 and 30 mg/kg, p.o.) reduced the number of lesions, with mirtazapine (30 mg/kg) having a greater effect than the two fluvoxamine doses indicated above.

In terms of biochemical parameters, Abdel-Hamed et al. (2022) found that mirtazapine was more effective than fluvoxamine in increasing the levels of PGE_2_, NO, GSH, SOD, and CAT while decreasing the amounts of myeloperoxidase (MPO) and MDA. In terms of gastric juice analysis, fluvoxamine and mirtazapine had similar effects, lowering stomach volume as well as total and free acidity levels. In this context, the authors conclude that mirtazapine is more gastroprotective than fluvoxamine since it blocks both norepinephrine and serotonin receptors at the same time.

Because of the previously reported hypothesis that the gastroprotective action of antidepressants is centrally mediated, Yao et al. (2022) sought to investigate the central mechanisms underlying the gastroprotective action of certain classes of antidepressants and their relationship with ASPA enzyme levels in the central nucleus of the amygdala in experimental models of stress-induced ulcers in male rats. Pre-treatment with fluoxetine (10 mg/kg, i.p.) significantly boosted ASPA enzyme levels, associated with decreased stomach lesions. The results are comparable to those reported by Yao et al. (2022) for an ADT. In this regard, the authors propose that the gastroprotective activity of fluoxetine is linked to the increased expression of ASPA in the central amygdala, and recommend that this enzyme be used as a novel target for stomach ulcer treatment research.

**Fig. 3:**
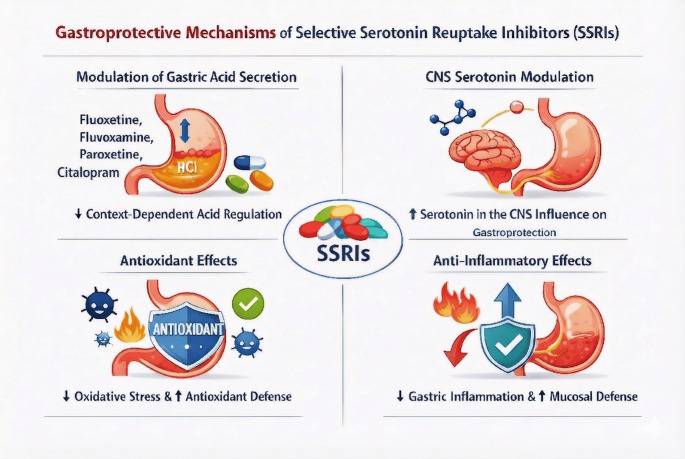
Gastroprotective mechanisms of selective serotonin reuptake inhibitors (SSRIs). SSRIs have gastroprotective effects through a variety of mechanisms, including altering stomach acid secretion, antioxidant activity, CNS impacts through increased serotonin availability, and anti-inflammatory effects. However, their effects can differ depending on the particular SSRI and context, necessitating careful consideration when using them

Evaluating the experimental outcomes is possible to infer that the gastroprotective effects of SSRIs are attributed to multiple mechanisms, including modulation of gastric acid secretion, antioxidant activity, and CNS influences (Fig. 3). However, some studies show that SSRIs reduce gastric acid secretion (Saxena and Singh 2011; Abdel-Hamed et al. 2022), while others suggest they may increase acid secretion and worsen gastric lesions (Takeuchi et al. 2011; Yamaguchi et al. 2008), depending on the context and specific SSRI used. SSRIs also exhibit antioxidant properties, reducing oxidative stress and enhancing antioxidant defenses in gastric tissue (Dursun et al. 2009a, b; Elsaed et al. 2018; Sokar et al. 2016). Additionally, increased serotonin availability in the central nucleus of the amygdala is proposed as a key mechanism underlying the gastroprotective effect of SSRIs, particularly against stress-induced ulcers (Ji et al. 2012; Yao et al. 2022), suggesting that the CSN plays a crucial role in mediating the gastroprotective effects of SSRIs. The anti-inflammatory effects of SSRIs contribute to their gastroprotective actions, reducing gastric inflammation and improving mucosal defense (Sokar et al. 2016; Abdel-Hamed et al. 2022). However, the effects of SSRIs on gastric ulcers can vary, highlighting the need for careful consideration in their use, particularly in combination with other medications like NSAIDs.

### Gastroprotective potential of antidepressants from other classes

Despite consistent findings on the gastroprotective activity of numerous antidepressants, the majority of which are tricyclic, there has been a lack of studies on the impact of atypical antidepressants. Suleyman et al. (2009) investigated the antiulcer activity of tianeptine, trazodone, and venlafaxine in male rats with indomethacin-induced ulcers. The study discovered that administering trazodone (12, 25, and 50 mg/kg p.o.) and venlafaxine (12, 25, and 50 mg/kg p.o.) did not prevent the formation of lesions in the gastric mucosa, in contrast to the results obtained with the use of tianeptine (6, 12, and 25 mg/kg p.o.), which were dose-dependent. The authors also stated that tianeptine’s activity was related to the regulation of antioxidant processes.

Bilici et al. (2008) investigated the antiulcerogenic properties of mirtazapine, as well as its association with putative oxidant and antioxidant pathways, in male rats with indomethacin-induced ulcers. After administering mirtazapine (15, 30, and 60 mg/kg p.o.), the authors noticed a dose-dependent reduction in the formation of lesions and hyperemia of the gastric mucosa, with the dose of 60 mg/kg showing to be the most efficacious among all tested. Furthermore, the study examined the effects of mirtazapine with famotidine, an inhibitor of histamine H2 receptors, and it was discovered that a dose of 30 mg/kg of mirtazapine had the same effectiveness as famotidine (20 mg/kg) in reducing injuries.

Furthermore, Bilici et al. (2008) found that all doses of mirtazapine increased GSH and SOD levels while decreasing CAT, MPO, and MDA levels, indicating an important antiulcerogenic effect via enzymatic and non-enzymatic activation of antioxidant mechanisms. Mirtazapine inhibits harmful processes, implying that it is a promising treatment for depressive individuals with stomach ulcers.

As previously stated, Ji et al. (2012) attempted to assess the effects of four antidepressants from distinct classes (duloxetine, amitriptyline, fluoxetine, and mirtazapine) in male rats exposed to various ulcer formation models. In the stress-induced model, duloxetine (5, 10, and 20 mg/kg, i.p.) and mirtazapine (10 and 20 mg/kg, i.p.) showed gastroprotective activity. Furthermore, at the aforementioned levels, duloxetine reduced intraluminal bleeding, but mirtazapine did so only at a dose of 20 mg/kg. Ji et al. (2012) propose that the increased availability of norepinephrine and serotonin is one of the mechanisms responsible for the gastroprotective action of these drugs, as earlier research demonstrated that the intracerebroventricular injection of serotonin and the injection of noradrenaline into the central nucleus of the amygdala was capable of attenuating the damage caused by stress-induced ulcers, indicating that this was primarily a CNS-mediated process.

Based on previous research linking antidepressant activity to antiulcerogenic properties, and given the scarcity of studies linking such effects to the class of monoamine oxidase inhibitors (MAOIs), Albayrak et al. (2014) investigated the gastroprotective action of moclobemide, a selective inhibitor of the enzyme monoamine oxidase-A (MAO-A), in male rats with indomethacin-induced ulcers.

Moclobemide (10, 20, 40, 80, and 150 mg/kg, p.o.) was efficient in reducing ulcerative lesions in the stomach mucosa, with the 20 and 40 mg/kg doses producing the most significant results. Furthermore, biochemical data revealed that the drugs were capable of enhancing antioxidant levels such as NO and SOD while decreasing oxidative stress levels such as MDA and MPO. Given the findings, the authors conclude that moclobemide has a significant gastroprotective effect, which is linked to the modulation of oxidant and antioxidant pathways (Albayrak et al., 2014).

Cao et al. (2020) found that administering duloxetine (5 and 20 mg/kg, i.p.) reduced bleeding sites in the stomach mucosa in a dose-dependent way. Using multiple tests, the scientists discovered that administering the medication at a dose of 20 mg/kg was adequate to inhibit the increase in MDA and oxidative protein levels in both induction models. Furthermore, this same dose was shown to be efficient in preventing the rise in NADPH oxidase activity that was observed following the administration of apocynin, indicating that duloxetine’s effects may be due to the same mechanism as the NOX inhibitor. Additionally, it was discovered that in the stress induction model, the administration of duloxetine at a dose of 5 mg/kg inhibited NOX1 expression, while 20 mg/kg inhibited NOX2, DUOX2, and NOX4. In the indomethacin model, SNRI at a dosage of 20 mg/kg reduced the expression of NOX1, DUOX2, and NOX4.

Cao et al. (2020) extended their analysis to moclobemide (20 mg/kg, i.p), an antidepressant that inhibits the enzyme monoamine oxidase, and found that in the stress model, NOX2 and DUOX2 levels were reduced, as were NOX1 and NOX4, whereas, in the indomethacin model, only NOX1 and DUOX2 were reduced.

In their quest for mechanisms of action, Yao et al. (2022) observed that pre-treatment with duloxetine (5 and 20 mg/kg, i.p) or moclobemide (20 mg/kg, i.p) raises ASPA enzyme levels. High levels of ASPA were associated with lower NAA levels and fewer stomach lesions, indicating that this is the primary molecular mechanism of gastroprotection not only for duloxetine but also for the other antidepressants tested in this study.

Atypical antidepressants are thought to have gastroprotective benefits through a variety of mechanisms, such as central nervous system influences, antioxidant activity, and gastric acid secretion control. According to certain research, atypical antidepressants like tianeptine may lessen the development of stomach lesions through antioxidant processes (Suleyman et al. 2009). Mirtazapine, duloxetine, and moclobemide possess antioxidant qualities that lower oxidative stress and strengthen antioxidant defenses in gastric tissue (Bilici et al. 2008; Albayrak et al., 2014; Cao et al. 2020). Furthermore, by modulating serotonin and norepinephrine tone, atypical antidepressants like duloxetine and moclobemide may have gastroprotective benefits through central pathways (Ji et al. 2012; Yao et al. 2022). Atypical antidepressants, including mirtazapine, have anti-inflammatory properties that contribute to their gastroprotective effects by lowering stomach inflammation.

## Perspectives

Given the results presented and the list of mechanisms of action involved in the antiulcerogenic activity of antidepressants, new studies are necessary to clarify certain aspects, such as the influence of gender on the gastroprotective action exhibited by the drugs, the discrepancy in data regarding the antiulcerogenic potential of paroxetine, as well as complementary analyses that evaluate the evolution of the healing process of existing ulcers, as all publications in this evaluation only looked at pre-treatment with the antidepressants studied.

The analysis of the risk of bias in the included studies highlights that the majority of domains were classified as having an unclear risk of bias, primarily due to insufficient reporting of details such as randomization and blinding procedures. Therefore, future studies on the gastroprotective effects of antidepressants should prioritize transparent reporting of their methodological details.

Furthermore, given the complex interplay between the gut-brain axis and the pathophysiology of gastric ulcers, it is essential that future studies investigate the role of this axis in the gastroprotective effects of antidepressants. Elucidating the mechanisms by which antidepressants interact with the gut-brain axis could provide valuable insights into their therapeutic potential and identify potential targets for the development of novel gastroprotective strategies. Therefore, future research should prioritize investigating the gut-brain axis as a key component of the gastroprotective effects of antidepressants, using approaches that integrate behavioral, neurobiological, and gastrointestinal outcomes.

## Conclusion

Taken together, experimental evidence shows that antidepressants are beneficial in preventing gastric ulcers and may be used for the development of new drugs to protect against gastric ulcer development in special situations. Antidepressants from various classes are capable of acting in the prevention of ulcers induced by different ulcerogenic agents, with a mechanism of action involving the modulation of oxidizing and antioxidant agents, as well as the modulation of the CNS, which is related to the suppression of vagal stimulus and, above all, the increase in noradrenergic and serotonergic neurotransmission.

## Supplementary Information

Below is the link to the electronic supplementary material.

Supplementary Material 1

## Data Availability

The protocol and review were not registered. Data collection forms, data extracted from included studies, data used for all analyses, and any other materials used in the review are available from the relevant author upon reasonable request.

## Abbreviations

ADT: Antidepressants
ASPA: Aspartoacyclase
ATP: Adenosine triphosphate
CAT: Catalase
CGRP: Calcitonin gene–related–peptide
COX−1: Cyclooxygenase−1
COX–2: Cyclooxygenase−2
DUOX: Dual oxidase 2
CNS: Central nervous system
GABA: Gamma–aminobutyric acid
GAD: Generalized anxiety disorder
GI: Gastrointestinal tract
GSH: Glutathione reduced
Hsp70: 70 kD heat shock protein
H1: Type 1 histamine receptor
H2: Type 2 histamine receptor
H2-RAS: Histamine receptor antagonists
i.c: Intracerebrally
i.p: Intraperitoneally
i.v: Intravenously
L-NAME: L–(G)–nitro–L–arginine–methyl–ester
LPO: Lipoperoxides
MAO-A: Monoamine oxidase A
MAOIs: Monoamine oxidase inhibitors
MDA: Malondialdehyde
MPO: Myeloperoxidase
M1: Type 1 muscarinic receptor
NE: Norepinephrine
NAA: N–acetyl–aspartate
NET: Norepinephrine transporter
NEM: N–ethylmaleimidine
NO: Nitric oxide
NOX: NADPH oxidase
NOX 1: NADPH oxidase 1
NOX 2: NADPH oxidase 2
NOX 4: NADPH oxidase 4
NSAIDs: Non–steroidal anti–inflammatory drugs
P-CABs: Potassium–competitive acid blockers
PGE_2_: Prostaglandin E_2_
PPIs: Proton pump inhibitors
PTSD: Post–traumatic stress disorder
ROS: Reactive oxygen species
SERT: Serotonin transporters
SOD: Superoxide dismutase
SH-NP: Non–protein sulfhydryl groups
SSRIs: Selective serotonin reuptake inhibitors
TCAs: Tricyclic antidepressants
TNF: Tumor necrosis factor
TRH: Thyrotropin–releasing factor
p.o: per os
5-HT: 5–hydroxytryptamine
