# Supplementary material for: Protective effects of antidepressants against ulcerogenic agents: insights from nonclinical studies
Source: Inflammopharmacology. 2026 Apr 22;34(5):2889–905. doi: 10.1007/s10787-026-02240-3 (PMC13179233; doi:10.1007/s10787-026-02240-3)
Supplement: Supplementary file 1 — Supplementary Material 1 [file 10787_2026_2240_MOESM1_ESM.docx]

**Protective Effects of Antidepressants Against Ulcerogenic Agents: Insights from Nonclinical Studies**

Caroline Stringari^1^, Heloisa Stringari^1^, Thiago Farias de Queiroz e Silva^2^, Caio Henrique Willrich^3^, Luisa Mota da Silva^3^*.

1. Pharmaceutical Sciences Graduate Program – University of Itajai Valley, Itajai, SC, Brazil.
2. Medicine course, Health school – University of Itajai Valley, Itajai, SC, Brazil.
3. Department of Pharmacology, Federal University of Santa Catarina, Florianópolis, Santa Catarina, Brazil.

*Corresponding author.

Tel.: +55 47 33415550

E-mail: [luisa.mota@ufsc.br](mailto:luisa.mota@ufsc.br)

**Figure 1SM.** Results from the analysis of the risk of bias


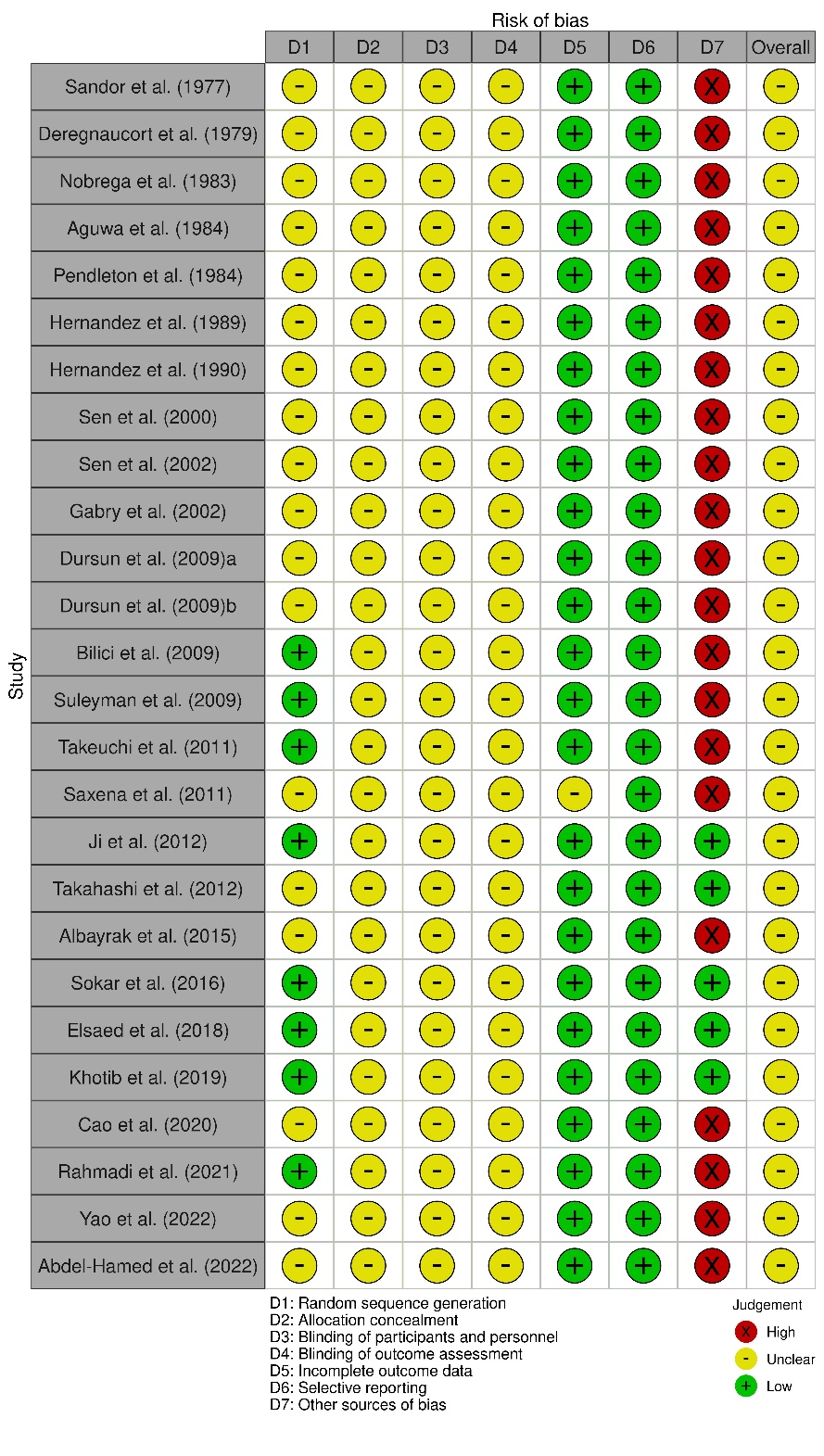


**Legend:** Risk of bias of the included studies, according to the assessed domains. Chart generated using the RobVis tool.
